# Challenges and prospects of yeast-based microbial oil production within a biorefinery concept

**DOI:** 10.1186/s12934-023-02254-4

**Published:** 2023-12-05

**Authors:** María Gallego-García, Ana Susmozas, María José Negro, Antonio D. Moreno

**Affiliations:** 1 Advanced Biofuels and Bioproducts Unit, Department of Energy, Research Center for Energy, Environment and Technology (CIEMAT), Avda. Complutense 40, Madrid, 28040 Spain; 2 Department of Biomedicine and Biotechnology, University of Alcalá de Henares, Alcalá de Henares, Spain

**Keywords:** Lignocellulose, Biowaste, Biofuel, Circular bioeconomy, Oleaginous yeast, Microbial oil

## Abstract

Biodiesel, unlike to its fossil-based homologue (diesel), is renewable. Its use contributes to greater sustainability in the energy sector, mainly by reducing greenhouse gas emissions. Current biodiesel production relies on plant- and animal-related feedstocks, resulting in high final costs to the prices of those raw materials. In addition, the production of those materials competes for arable land and has provoked a heated debate involving their use food vs. fuel. As an alternative, single-cell oils (SCOs) obtained from oleaginous microorganisms are attractive sources as a biofuel precursor due to their high lipid content, and composition similar to vegetable oils and animal fats. To make SCOs competitive from an economic point of view, the use of readily available low-cost substrates becomes essential. This work reviews the most recent advances in microbial oil production from non-synthetic sugar-rich media, particularly sugars from lignocellulosic wastes, highlighting the main challenges and prospects for deploying this technology fully in the framework of a Biorefinery concept.

## Introduction

The development and implementation of a circular bio-based economy have been extensively promoted in recent years as an alternative to the use of fossil resources. This transformative process will definitely contribute to the UN’s Sustainable Development Goals, involving a wide range of economic sectors and industries (e.g., food, forestry, pharmaceuticals, chemistry and textiles) [1]. Among the diverse bioproducts available, biofuels have a central role in developing such a bioeconomy, and extensive research efforts have been targeted at placing these compounds on the market. Independently of the raw material from which biofuels are produced, these bio-based products can enable energy independence, reduce greenhouse gas emissions, and enhance sustainable economic development [2]. In addition, costs can be decreased by the use of residual products. Among the different residues, low-input farming have the smallest carbon and nitrogen footprints and are usually cheaper feedstocks.

Biodiesel is the second most-produced liquid biofuel after bioethanol worldwide, reaching 48 billion litres in 2021 [2]. Current biodiesel production is based on vegetable oils and animal fats. These raw materials are usually associated with limited availability, competition with arable land and high costs. In contrast, single-cell oils (SCOs, also known as microbial lipids) obtained from oleaginous microorganisms have been positioned as a promising alternative for intermediate hydrocarbon biofuel production. In addition to having a similar composition to vegetable oils, these feedstocks do not depend on geographic location, seasonal changes, harvest schedules or transportation, and they usually exhibit fast production rates and are easy to scale up for industrial processing [3, 4]. However, large-scale biodiesel production from microbial oils still requires overcoming some obstacles, including harsh microbial culture conditions and difficulties during microbial harvesting and oil extraction [5].

Oleaginous microorganisms are bacteria, yeasts, filamentous fungi, and microalgae that may accumulate lipids intracellularly with concentration of over 20% (w/w) of their dry weight and, under special condition, up to 80% (w/w) [5–7]. These microorganisms may grow in a wide range of substrates, from pure glucose to organic wastes, the latter the preferred choice due to the possible cost reduction [8]. In addition to the lower prices, the use of wastes for microbial oil production will reduce the overall discarded waste figures, thus contributing to the zero-waste target of the circular economy.

Oleaginous microorganisms can also co-produce compounds with biotechnological potential in a biorefinery process. Therefore, besides obtaining SCOs, the process may benefit from recovering other high-added-value products that can make production more economically attractive. These compounds include citric acid, carotenoids, biosurfactants, glycerol and mannitol, among others [9–12]. The present article highlights the recent advances in using low-cost, non-synthetic sugar-rich media, including lignocellulosic residues and industrial wastes for SCO production, targeting and discussing the main achievements in the case of microbial lipid production by yeasts. In addition, the most promising co-products will be listed and critically discussed from an integrated biorefining perspective.

## Microbial oil production: process overview and cultivation strategies

To reduce the impact of scaling up production and to increase the chances of placing SCO-based biofuel on the market, developing robust bioprocesses is key [13].

Productivity in terms of high concentrations generated in relatively short periods of time is one of the most critical considerations in microbial oil production. Furthermore, an oil content of 40% has been set as the threshold-accumulated amount to reduce waste production during downstream processing [14]. Optimising process parameters and designing effective fermentation strategies have been the main targets for improving the conversion of raw materials into SCO [15].

Yeast cells trigger lipid production mainly under nutrient-limiting conditions and in the presence of high carbon/nitrogen (C/N) ratios [5, 16, 17]. Nitrogen, sulphur and phosphorus are the main nutrient-limiting factors used to activate lipogenesis. However, low concentrations of a specific nutrient usually result in low cell biomass and may even cause cell growth inhibition [18]. André et al. reported a maximum cell biomass concentration of 4.2–8.2 g/L dry cell weight (DCW) when the yeast *Yarrowia lipolytica* was grown on a medium having crude glycerol (30 g/L) and ammonium sulphate and yeast extract (0.5 g/L each) as major carbon and nitrogen sources. Although the lipid accumulation found was low (maximum 0.20 g lipids/g DCW), non-negligible amounts of citric acid and mannitol were found in the growth medium. [19]. Similarly, *Rhodotorula glutinis* reached a cell biomass of 5.3 g/L when grown in pure glycerol at about 700 C/N ratio [20]. Glucose-based media have also yielded low cell biomass concentration (4.5 g DCW /L) when using *Y. lipolytica* at a C/N ratio of about 75 [21]. High lipid accumulation (47.5 w/w) was obtained using *Y. lipolytica* in a glucose-based media limited in nitrogen and magnesium [22]. Subsequently, by applying an adaptive laboratory evolution strategy of the same strain used in the work, as mentioned earlier, it was possible to increase the lipid content by 30%. The evolved strain was obtained after 77 generations in lack of nitrogen and magnesium while using glucose as a carbon source [23]. Final cell biomass concentrations are directly linked to the final microbial oil concentrations. Hence, final cell biomass concentrations of about 4.5–6.5 g/L DCW can result in up to 2 g/L of lipids, with oil content ranging from 30 to 45% [21–23]. *Y. lipolytica*, has also shown the capacity to accumulate endo-polysaccharides in the early stages of culture despite the presence of nitrogen in the medium [24]. This interesting trait has also been reported in other yeast such as *Rhodosporidium toruloides, Cryptococcus curvatus* and *Lipomyces starkeyi*. These endo-polysaccharides can be therefor considered as advantageous co-products increasing the economic viability and sustainability of the SCO bioprocess [25–27].

In contrast to the batch operational mode, using fed-batch and continuous cultivation modes can improve substrate utilisation, thus contributing to higher conversion yields [28, 29]. These alternatives favour greater productivity and prevent the systems from inhibiting cell growth. Karamerou and Webb have reviewed how these operational modes influence the process to provide the necessary nutrients during each growth phase and support high lipid production titers [13].

In fed-batch culture, the substrate is fed to the system at different stages during the cultivations phase, while the product remains in the bioreactor until the end of the run. Two common fed-batch strategies are used for SCO production: (1) fed-batch cultivations with pulse-feeding medium addition and (2) fed-batch cultivations with continuous medium supply. Regardless of the strategy use, fed-batch cultivation effectively increases final cell biomass and lipid concentrations [30]. For instance, a fed-batch strategy using a glucose medium increased the cell biomass concentration of *Candida viswanathii* from 17 g/L DCW with the batch strategy to 21 g/L DCW [31]. In addition to the higher final cell biomass concentration, these authors reported an increase in the oil content from 33 to 50%, doubling the final lipid concentration (from 5.6 g/L to 10.5 g/L). Raimondi et al. also increased the final oil concentration obtained with the yeast *Candida freyschussii* using a fed-batch strategy with pure glycerol media [32]. In that study, 2-pulse, 4-pulse and continuous fed-batches increased SCO content 2.0-, 4.4- and 6.1-fold, respectively, for final lipid concentrations of 9.1 g/L, 20 g/L and 28 g/L compared to the 4.6 g/L concentration obtained with the batch strategy. In contrast to the study with *C. viswa*nathii which increased its intracellular oil content by 1.5 times [31], *C. freyschussii* always produced an oil content in the range of 30–35% independent of the operation mode and the feeding strategy [32].

The fed-batch operation mode with a pulsed medium addition strategy can follow different feeding patterns based on parameters such as a specific nutrient concentration (commonly the carbon source), the dissolved oxygen levels and the fermentation time [20, 33–35]. One of the main approaches used for the further addition of the carbon source is when this nutrient is at low levels. Maina et al. fed the system with a glucose solution when the concentration of this component decreased to around 5 g/L [33]. A similar strategy was followed by Thiru et al., where the system was fed with a concentrated glycerol solution when the concentration was below 3 g/L [34]. This feeding strategy contributes to increased final cell biomass concentrations. For instance, a final cell biomass concentration as high as 110 g/L DCW was obtained by Tsakona et al. when growing *Lipomyces starkeyi* in a flour-rich hydrolysate using pulse addition when glucose content dropped below 20 g/L [36]. The relative dissolved oxygen level is another parameter that can be used to design the feeding strategy. Dissolved oxygen content increases when the metabolic activity of the fermentative microorganism decreases, mainly due to carbon source depletion. When observing such a shift in the system, the carbon source is then fed accordingly. This strategy was followed by Meesters et al. using *Cryptococcus curvatus* as the fermentative microorganism and 87% pure glycerol as the carbon source [37]. Although very high final cell biomass concentrations of up to 118 g/L were obtained, the authors reported a maximum oil content of only 25%. In addition to monitoring the carbon concentration and oxygen levels, the feeding strategy can be done by simply considering a specific time interval. *R. glutinis* was cultured in a glycerol-based medium following a fed-batch strategy where the fresh substrate was added to the system every 24 h, almost doubling the final cell biomass concentration (9.4 g/L vs. 5.3 g/L) and the lipid content (2.6 g/L vs. 1.7 g/L) [20]. Uçkun Kiran et al. also followed this process scheme with *Rhodosporidium toruloides* using crude glycerol as the carbon source [38]. After a batch phase of 72 h, crude glycerol was added to the system every 24 h to achieve final concentrations of 70–90 g/L. This approach produced a final cell biomass concentration of 23.1 g/L and a lipid content of 9.4 g/L, compared to 12.1 g/L and 6.1 g/L, respectively, obtained in the batch mode.

As an alternative to the pulse-feeding strategy, the substrate can be fed to the system continuously. With the continuous fed-batch strategy, finding the optimal feeding rate is of utmost importance for effective lipid production, although only a few studies have addressed this point [13, 31, 32, 39]. For instance, Zhao et al. designed a continuous feeding strategy to keep the glucose concentration at low levels (< 5 g/L) during the feeding phase [39]. This process scheme increased the cell biomass concentration and oil content from 89 g/L DCW and 52.2% to 127.5 g/L DCW and 61.8% compared to a pulsed-feeding strategy. In contrast, Raimondi et al. followed a continuous feeding strategy using glycerol as the carbon source, which resulted in glycerol accumulation in the medium at the initial stages of substrate addition [32]. However, it also produced a higher cell biomass concentration when compared to the pulsed-feeding strategies. Other alternatives to the constant feeding of the substrate for SCO production are exponential and variable feeding, where the feeding rate is adjusted according to specific equations or to maintain a particular parameter (e.g. C/N ratio) [40, 41]. These strategies have also resulted in high cell biomass concentration with a high accumulation percentage. For example, fed-batch cultivation of *R. glutinis* with exponential feeding resulted in a final cell biomass concentration of about 40 g/L with an oil content of 43% [41]. On the other hand, a final cell biomass concentration as high as 132 g/L DCW with an oil content of about 55% was observed when culturing *Trichosporon oleaginosus* with a feeding strategy based on maintaining a constant C/N ratio [40].

During fed-batch fermentation, it is essential to consider the dilution effect to avoid reducing cell density and to keep nutrient concentrations at adequate levels [13]. In this context, using concentrated substrate solutions containing all required nutrients during the feeding phase is essential [32].

Continuous cultivation in SCO production is also of interest because it increases productivity compared to the batch fermentation mode [18]. This system continuously provides carbon and nitrogen sources while allowing continuous collection of products and cells. A constant C/N ratio is preserved when reaching the steady state phase at a specific dilution rate (D). Under these conditions, cell concentration remains constant since growth and substrate uptake rates do not vary, while lipid production is promoted by controlling the nitrogen concentration. The dilution rate is the main parameter affecting the final cell biomass concentration and lipid content. In this regard, Papanikolaou and Aggelis observed a reduction in the lipid content from 3.5 to 0.3 g/L and in the cell biomass concentration from 8.1 to 3.8 g/L after increasing the dilution rate from 0.03 h^-1^ to 0.13 h^-1^ during the continuous cultivation of *Y. lipolytica* LGAM S(7)1 in crude glycerol [42]. Similar results have also been found when using other carbon sources and microbial strains. For instance, continuous fermentation of *R. toruloides* AS 2.1389 in glucose media at D ranging 0.02–0.20 h^-1^ resulted in cell biomass concentrations of 1.63–8.67 g/L DCW and lipids content of 0.21–5.36 g/L, the lower values being obtained with the higher dilution rates [43]. Similarly, cell biomass concentration and lipid content in *C. curvatus* decreased from 5.1 to 0.8 g DCW/L and 3.4 to 0.12 g lipid/L after increasing D values from 0.01 to 0.11 h^-1^ in an acetate-based medium [44].

In addition to affecting lipid production, the feeding rate in continuous cultivation is critical in directing carbon flux and cellular metabolism to energy, maintenance and product synthesis [42, 45]. This system benefits from continuously harvesting cells that can be processed directly after collection. Nevertheless, long-term fermentation can suffer from media sedimentation and biofilm formation. Furthermore, there is a high risk of contamination, and the yield can fluctuate depending on cellular changes [15].

Other advanced cultivation systems have been developed to optimise the process of SCO production. One such system is two-stage fermentation, which first targets producing cell biomass in nutrient-rich conditions and then on lipid accumulation under nutrient limitations and in an excess of carbon [46]. Therefore, the two-stage lipid production process focuses seeks to improve lipid production by optimising both cell proliferation and lipid accumulation stages. The accumulation stage requires optimisation when using this cultivation system, since cell growth only requires nutrient-rich media. In this context, these cultivation systems can follow a two-stage batch or two-stage strategy with a feeding addition [45, 47].

## Lignocellulosic biomass and industrial wastes as raw material

During SCO production, the cost of raw materials can account for 40–70% of the total overall costs, with highly refined polysaccharides such as industrial-grade glucose and sucrose accounting for about 80% of such costs [47–51]. Using low-cost substrates, including lignocellulosic biomass and industrial waste, are promising alternatives for microbial lipid production [52–54].

Residual lignocellulosic biomass and waste-derived substrates are excellent candidates as carbon sources to make this process both cost-effective and environmentally friendly. In this context, sugarcane bagasse residues, cheese whey, corn stover, potato wastewater or orange peel extracts have been previously investigated for SCO production [55–59]. Table 1 summarises the most recent literature on using biomass residues for SCO production, mainly focusing on lignocellulosic sources.

Oleaginous yeasts can also use hydrophilic and hydrophobic substrates to accumulate lipids via two pathways: *de novo* and *ex novo* lipid synthesis [60]. Notably, both pathways offer distinct advantages: *de novo* lipid fermentation can generate a high quantity of lipids. In contrast, *ex novo* lipid fermentation can modify the lipid compositions to satisfy the requirements of the chemical or food industries. Thus, improving and upgrading fatty materials utilized as substrates can produce “tailor-made” lipids of high-added value [61]. The combined production of *de novo* and *ex novo* lipids has been studied, for instance, by using the yeast *Trichosporon dermatis* and a mixed medium combining an acid hydrolysate of corn cob and a soybean. That study confirms the potential of using both substrates (hydrophilic and hydrophobic) for the production of lipids with application in the production of biodiesel or lipid-based chemical compounds. [62].

**Table 1:** Recent literature on lipid production by oleaginous yeast from lignocellulosic biomass

Microorganisms	Lignocellulosic raw material/Pretreatment	Cultivation mode	Lipid content (w/w)	Lipid Yield Yp/s	Lipid concentration	Reference
*C. curvatus*	Rice straw Pretreatment (Glycerol-FeCl_3_)	Batch	46.8%	0.17 g lipid/g	8.8 g/L	[63]
*C. curvatus* DSM 70022	Wastepaper Oxidising pretreatment (hydrogen peroxide 120ºC-30 min)	Batch (Flask Erlenmeyer)	37.8%	0.234 g lipid/g	5.75 g/L	[64]
*C. curvatus* DSM 70022	Wastepaper pretreated (diluted acid)	Batch (Flask Erlenmeyer)	43.1%	0.141 g lipid/g	4.95 g/L	[64]
*Cryptococcus sp MTCC 5455*	*Brassica juncea* Microwave-Assisted Dilute Alkali Pretreatment	Batch (Flask Erlenmeyer)			11.05 g/L	[65]
*C. oleaginosum*	Corn stover Alkali organosolv pretreatment (NaOH:Methanol, 80 ºC, 1 h)	Batch (Erlenmeyer Flask) Fed-batch (2 pulses)	61.7%	0.18 g lipid/g	31.3 g/L	[66]
*L. starkeyi*	Sugarcane bagasse (hydrolysate) 1.5% sulfuric acid (w/v) at a solid-to-liquid ratio of 1:10 in an autoclave at 120 °C for 20 min	Batch (Erlenmeyer Flask) Bioreactor (3 L) Continuous	26.1% 27.3%	0.14 g lipid/g 0.18 g lipid/g	2.1- g/L 3.14 g/L	[67]
*L. starkey DMS 90276*	Sugarcane bagasse (hemicellulose hydrolysate) Pretreatment dilute acid (1.5% (w/v) H_2_SO_4_ at 120 C for 20 min)	Batch (Erlenmeyer Flask)	44.8%	0.16 g lipid/g	3.53 g/L	[68]
*L. starkeyi DSM 70295*	Oil palm empty fruit bunch Alkali pretreatment (3.75 M NaOH, 120º C, 1 h)	Batch (Erlenmeyer Flask)	40%	0.162 g lipid/g	4.9 g/L	[69]
*L. starkeyi* NRRL Y-11557	Corn stover Ammonia fiber explosion (AFEX) pretreatment	Batch (Erlenmeyer Flask)	38%	0.14 g lipid/g	9 g/L	[70]
*M. pulcherrima*	Starch hydrolysate	Batch fermentation (250 L)	34.3%	0.21 g lipid/g	15.8 g/L	[71]
*M. pulcherrima*	Starch hydrolysate	Semi-continuous (250 L)	32.6%	0.16 g lipid/g	11.6 g/L	[71]
*R. babjevae DVBPG 8058*	Wheat straw hydrolysate (steam explosion 190ºC 10 min; 1% acetic acidic (soaked overnight)	Batch (0.5 L Bioreactor)	64.8%	0.24 g lipid/g	18.1 g/L	[72]
*R. fluvialys*	Sugarcane top (alkaline-hydrogen pretreatment)	Bath	63.1%	0.23 g lipid/g	19.1 g/L	[73]
*R. fluvialys*	Sugarcane top (alkaline-hydrogen pretreatment) plus crude glycerol	Fed-batch (3 L fermenter) (continuous fed crude glycerol solution)	61.4%	0.33 g lipid/g	23.6 g/L	[73]
*R. glutinis*	Corncob Acid pretreatment (sulfuric acid + phosphoric acid, S/L ratio 1/3, 123ºC 60 min)	Batch (5 L fermentor) High cell density culture with two-stage nitrogen feeding strategy	47.2%	0.159 g lipid/g	33.5 g/L	[74]
*R. glutinis +* *C. pyrenoidosa*	Cassava bagasse enzymatic hydrolysate (concentrated)	Batch (Erlenmeyer Flask)	58.73%	0.230 g lipid/g	17.7 g/L	[75]
*R. taiwanensis*	Corncob hydrolysate (sulphuric acid 2.5% 121 2.5 h) detoxified	Batch (5 L fermenter)	60.3%	0.056 g oil/g corncob	11.2 g/L	[76]
*R. toruloides*	Maple Wood Alkali pretreatment (NaOH 1%, S/L 1/10 (w/v) 121ºC 15 psi, 30 min)	Erlenmeyer	36.68%	n.a.	6.25 g/L	[77]
*R. toruloides AS 2.1389*	Corn stover Dry acid pretreatment and biodetoxification (2.5 g H_2_SO_4_/100 g dry corn stover; 175 °C, 5 min followed by biological detoxification process with *Amorphotheca resinae*)	Flasks SSLP (simultaneous saccharification lipid production) PSSLP (prehydrolysis SSLP)		0.062 g/g corn stover 0.08 g/g corn stover 0.077 g/g corn stover	6.2 g/L 10.1 g/L 11.4 g/L	[78]
*R. toruloides CBS 14*	Wheat straw Acid-based steam explosion hydrolysate (10%) + crude glycerol	Batch (0.5 L Bioreactor)	46.8%	0.25 g lipid/g	10.6 g/L	[79]
*R. toruloides CBS 14*	Wheat straw Hydrolysate (steam explosion 190ºC 10 min; 1% acetic acid (soaked overnight)	Batch (0.5 L Bioreactor)	39.31%	0.15 g lipid/g	11.72 g/L	[72]
*R. toruloides CBS 14*	Wheat straw Steam explosion (Acid-soaked (1% acetic acid), steam explosion 190 °C, 10 min.)	Batch (2 L Bioreactor)	41%	0.13 g lipid/g		[80]
*R. toruloides DSMZ 4444*	Corn stover (Combined pretreatment) NaOH 80ºC 2 h)/sulfuric acid preimpregnation 2 h, 160ºC-10 min )	Batch DO-Fed-batch Pulse-Fed-batch Online-FB	56.67% 59.81% 61.54% 58.76%	0.19 g lipid/g 0.23 g lipid/g 0.24 g lipid/g 0.29 g lipid/g	21.2 g/L 25.2 g/L 26.7 g/L 31.7 g/L	[ [81]
*R. toruloides* NRLL	Fir wood (NaOH 1%, S/L 1/10 (w/v) 121ºC 15 psi, 30 min)	Batch (Erlenmeyer Flask)	35.24%	n.a.	6.88 g/L	[77]
*R. toruloies CCT 7815*	*Eucalyptus urograndis* Hemicellulose hydrolysate, 160ºC 195 min	Batch (Erlenmeyer Flask)	50%	0.13 g lipid/g		[82]
*Trichosporium dermatis*	Dilute acid corn stover (1% w/w H_2_SO_4_ 160ºC-10 min), pretreated biomass without washing. (1%H_2_SO_4_ 180ºC-10 min), pretreated biomass with washing.	Batch (Erlenmeyer Flask)	24.23% 45.06%	0.104 g lipid/g 0.156 g lipid/g	7.46 g/L 11.43 g/L	[83]
*T. dermatis*	Alkali pretreated corn stover (2% w/w, 120 ºC, 20 min -pretreated biomass without washing -pretreated biomass with washing	Batch (Erlenmeyer Flask)	28.4% 55.97%	0.101 g lipid/g 0.186 g lipid/g	6.81 g/L 20.36 g/L	[83]
*T. oleaginosus*	Shorghum stalk Swithgrass Alkali pretreatment (10%, w/v loading solid, 1.25%, w/v NaOHa, 121ºC, 30 min, 1 h)	Batch (Erlenmeyer Flask)	60% 58%	0.29 g lipid/g 0.27 g lipid/g	13.1 g/L 12.3 g/L	[84]
*Y. lipolytica*	Grass *Cyperus distans* Hydrothermal pretreatment (200ºC 60 min plus delignification with diluted alkaline)	Batch (5 L bioreactor)	53.62%	n.a.	10.58 g/L	[85]

n.a., not available

For SCO production, lignocellulosic biomass feedstock processing is required to obtain the corresponding fermentable sugars. This processing step depends a great deal on the biomass. Sugars contained in non-lignocellulosic wastes, such as agri-food residues, can be easily obtained by crushing and centrifugation processes [48, 86, 87]. In contrast, lignocellulosic biomass is highly recalcitrant to hydrolysis, and biomass pretreatment is required to alter its structure and increase the accessibility of hydrolytic enzymes to the sugar polymers before the saccharification step. A wide range of pretreatment methods using physical, chemical, physicochemical or biological approaches has been investigated towards this aim. Pretreatment processes have been extensively reviewed to summarise and update the main most important new developments and features from each technology. There is no universal pretreatment technology that can be applied to lignocellulose, since biomass is a heterogeneous category including materials that are sufficiently distinct as to require different approaches [88]. In general, biomass pretreatment usually requires severe conditions, such as high temperatures (> 160 °C), high pressures (5–30 bar) and/or the addition of an acid/alkali catalyst [89]. These conditions lead to biomass degradation and the generation of certain inhibitory compounds that limit the subsequent saccharification and fermentation steps. The concentration and nature of these inhibitors are directly correlated with the raw material (i.e., herbaceous biomass, hardwood, softwood), the pretreatment technology and the conditions used (temperature, use of catalysts, the residence time, pH, pressure). Inhibitors are classified into weak organic acids, furan derivatives, and phenolic compounds. The inhibition mechanisms of these compounds differ between these groups, and are usually highly interactive and synergistic, thus limiting biomass conversion processes by affecting both hydrolytic enzymes and fermentative microorganisms strongly. Different detoxification methods and/or increasing cell robustness of fermentative microorganisms have been investigated to overcome such inhibitory effects. Traditionally, pretreated biomass has been separated into liquid and solid fractions, and the solids are further subjected to a washing step before enzymatic hydrolysis to allow conversion of that sugar-rich fraction. By washing the resulting pretreated solid fractions, Yu et al. increased lipid yield from dilute acid pretreated and dilute alkali pretreated corn stover about 1.5-fold and 1.8-fold (from 0.101 to 0.104 g/g to 0.156 and 0.186 g/g), respectively [58].

In addition to washing, several detoxification methods have been tested to reduce the inhibitory potential of lignocellulosic pretreated substrates. Major detoxification methods are classified into chemical treatment (e.g., overliming, surfactant addition), adsorption methods (e.g., resins, activated carbon) and biological treatment processes (e.g., enzyme and microbial treatment) [90]. In situ biodetoxification strategies are attractive approaches compared to other detoxification methods due to the cost reduction possible. In this context, increasing cell robustness against lignocellulosic-based inhibitors will provide novel microorganisms that can tolerate higher concentrations of these compounds [71]. For instance, the adaptation of *R. toruloides* was successful in increasing tolerance to inhibitors present in sugarcane hydrolysates and lipid production as compared to the parental strain [52]. Liu et al. subjected *R. toruloides* to evolutionary engineering using a wheat straw hydrolysate, increasing its growth performance in a highly challenging medium [91].

According to the recent literature (Table 1), the yeasts *C. curvatus* [63–65, 92, 93], *L. starkeyi* [67–70], *Metschnikowia pulcherrima* [71], *Y. lipolytica* [85]; and species belonging to the *Rhodosporidium* and *Rhodotorula* genera [72–74, 76–82, 94] are the main microbial strains being used for microbial lipid production with lignocellulosic biomass residues. These yeast strains have been tested for lipid production using different raw materials, including agricultural wastes (e.g., corn stover, wheat/rice straw, sugarcane bagasse), woody residues (e.g., maple wood, fir wood, eucalyptus) and energy crops (e.g., switchgrass) pretreated with a wide range of technologies including alkali/acid pretreatment, organosolv, oxidising pretreatment and ionic liquids, among other methods. In these studies, the intracellular lipid content, the lipid concentration and the lipid yields ranged from 2 to 65%, 2–33 g/L and 0.06–0.33 g lipid/g substrate, respectively. The highest intracellular lipid content using native oleaginous yeast strains has been reported in 60–65% (w/w) [72]. Brandenburg et al. reported a total intracellular lipid content of about 64.8% (with a total lipid production of 18.1 g/L and a conversion yield of 0.24 g/g) using a steam-pretreated wheat straw hydrolysate (steam explosion 190ºC, 10 min; impregnated with 1% acetic acid) and the yeast *Rhodotorula babjevae* DVBPG 8058 [72]. Poontawee et al. estimated an intracellular lipid content of 63.1% in the yeast *Rhodosporidium fluvialys* using sugarcane top biomass pretreated by alkaline-hydrogen pretreatment [73]. A total lipid content of about 60% has also been accumulated by the yeasts *Cutaneotrichosporon oleaginosum*, *R. toruloides*, *Rhodotorula taiwanensis* and *Trichosporon oleaginosus*, with yields ranging from 0.056 to 0.29 g/g [66, 76, 81, 84].

Despite previous examples showing very high lipid content, this parameter in non-genetically modified strains usually accounts for up to 30–40% of the total CDW. Cell biomass production and intracellular lipid content can be increased by co-utilising different substrates. For instance, crude glycerol (a by-product obtained during the transesterification reaction from biodiesel production) has been combined with different hemicellulosic hydrolysates obtained from herbaceous and woody biomass. Chmielarz et al. used *R. toruloides* to ferment a mixture of steam-exploded wheat straw hydrolysate and crude glycerol, resulting in shorter fermentation times [79]. A total lipid titer of 10.6 g/L with lipid yields of 0.25 g lipids/g consumed carbon was observed. Saini et al. combined a woody hemicellulosic hydrolysate with crude glycerol in a 60:40 ratio, which resulted in the consumption of 90% of the sugars and glycerol from the media and reporting a maximum intracellular lipid content of 56.3% (w/w) [95]. In that work, biomass and lipid volumetric productivity values were 0.28 g/L h and 0.15 g/L h, respectively. Furthermore, these authors reported a reduction in the fermentation time of about 50%, which represents a significant advantage for the process, as it saves energy and ultimately lowers total production costs.

The internal recycling nutrients and wastewater effluent for subsequent cultures represents an attractive strategy to improve both the economic viability and sustainability of bioprocessing for microbial oil production. For example, this concept has been applied to improve lipid production by recycling spent cell mass and lipid fermentation wastewater in microbial oil production from corn stalk hydrolysates obtained after alkali pretreatment by *R. toruloides* Y4. Results obtained in that study showed that up to three cycles could be applied in fully recycling the resources with only slightly reduced lipid production [96]. In addition to recycling the resulting wastewater internally during lipid production, the composition of this resource makes it appropriate to further use it in other bioprocesses to produce high-value products. For instance, the fermentation broth obtained after lipid production, during the fermentation of a corncob acid hydrolysate by *Trichosporon cutaneum*, which mainly contained the residual sugars and extracellular polysaccharides, was successfully used as substrate for bacterial cellulose production by *Gluconacetobacter xylinus* [97]. In addition, the wastewater resulting from lipid fermentations has been also used as a maceration water for the production of edible and medicinal mushrooms within a biorefinery concept [98].

An essential factor that must be considered during SCO production is the co-production of CO_2_ during the process. The production of CO_2_ may account for up to 35–50% (w/w) of the carbon in the raw material. A smart solution that has been proposed to reduce these values is the capture of CO_2_ by integrating the production of lipids with microalgae cultivation [99]. Liu et al. investigated using a mixed culture between *R. glutinis* and *Chlorella pyranoidosa* to produce lipids from an enzymatic hydrolysate obtained from cassava bagasse [75]. This co-culture strategy was also combined with a fed-batch cultivation mode, obtaining promising results in terms of lipid concentrations (18.47 g/L), intracellular content (58.73% w/w) and lipid yields (0.23 g/g of consumed sugar). Similarly, Zuccaro et al. investigated a mixed culture using *Chloroidium saccharophylum* and the yeast *L. starkeyi* for lipid accumulation [100]. Among the benefits of this co-culture is the high capacity of *C. saccharophylum* to assimilate CO_2_, its ability to grow under heterotrophic conditions, and its high tolerance to acidic environments, together with the ability of *L. starkeyi* to catabolise the inhibitors that are present in the lignocellulosic hydrolysates. Despite the potential of using such co-culture, the enzymatic hydrolysate obtained from steam-pretreated *Arundo donax* as medium (steam explosion: 210ºC for 4 min; enzymatic hydrolysis: 5% substrate loading, 50ºC, 15 FPU cellulases/g cellulose; 30 CBU beta-glucosidase/g cellulose) resulted in low final lipid concentrations and lipid yields when compared to the results obtained with other consortia [75]. These authors attributed the lower final yields to a low sugar concentration during the experimental assays.

## The biorefinery concept: taking advantage of co-production possibilities

**Fig. 1:**
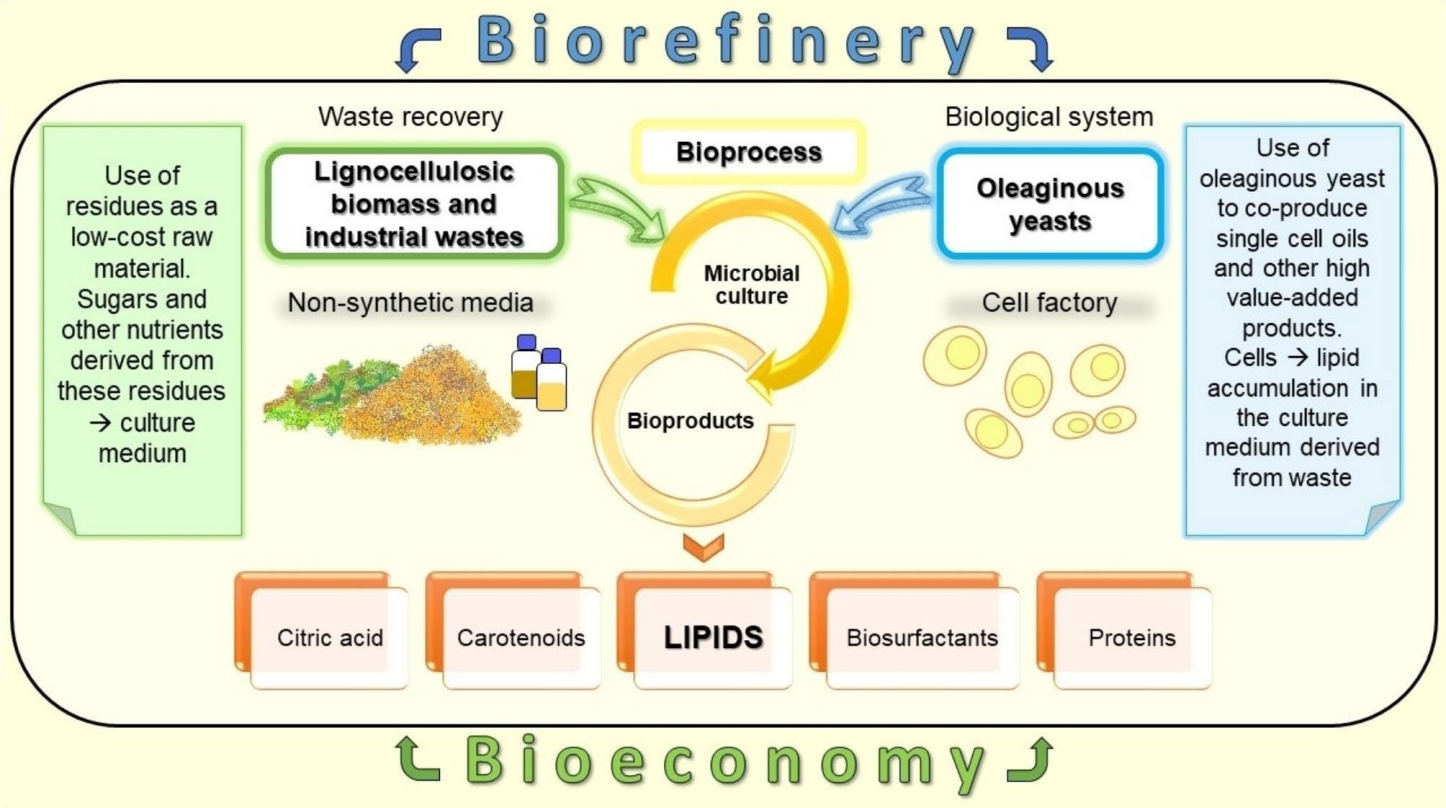
Yeast cell factory for microbial oil production and value-add co-products

In addition to using low-cost substrates, the economics of SCO production may also benefit from obtaining co-products of interest to industry, such as citric acid, carotenoids, biosurfactants, glycerol or mannitol [9–12, 48, 101] (Fig. 1). Yeasts capable of accumulating lipids and co-producing carotenoids, enzymes and other functional products have been considered excellent candidates for their potential use in biorefineries [102–104]. Among these products, carotenoids have been recognised as attractive food additives and nutritional supplements. Some carotenes (e.g., β-carotene) can be used as antioxidants, protecting cells, tissues and organs from the damaging effect of free radicals, thus preventing the development of some disorders such as cancer and/or heart disease [105, 106]. In 2022, the global β-carotene market reached $575 million, which is expected to increase to $955 million by 2032 [107]. Carotenoids are currently obtained from plant materials through extraction processes, microbial-based biosynthesis, and chemical synthesis [108]. More than 90% of commercial carotene is produced by chemical synthesis, which has been criticised for the high toxicity of the reagents used and the formation of unwanted by-products.

To obtain these products more sustainably and meet the increasing demand, new emerging technologies are under investigation. Therefore, using microbial cells as biocatalysts for carotenoid production represents an attractive approach for achieving this goal. This strategy also offers additional advantages, including a short and environmentally friendly production cycle. Yeasts belonging to *Rhotodotorula* species have exhibited the ability to produce carotenoids such as β-carotene, γ-carotene, torularhodin and torulene, and oil-lipids with an extensive profile of fatty acids. All these products can be obtained from a wide range of substrates, including lignocellulosic residues and industrial wastes such as sisal bagasse, sugarcane bagasse, corncob, wheat bran, switchgrass and wheat straw, camelina waste [91, 104, 109–113] (Table 2).

Yeasts produce carotenoids as antioxidant molecules to protect them from the cell damage resulting from the intracellular formation of reactive oxygen species (ROS), which are generated under stress-related conditions [114]. The kinetic parameters for carotenoid production are not only dependent on the yeast strain used but also on the specific culture conditions. As with the lipid production processes, different optimisation strategies have been tested to maximise carotenoid production [103]. The synthesis of carotenoids is influenced by factors involving microbial growth, including sugar concentration, temperature, pH, aeration rate and salt addition. Being an aerobic process, aeration and/or oxygenation is essential for carotenogenesis. In addition, culture supplementation with salts can stimulate the metabolic pathway of carotenoid-synthesising enzymes [112]allowing, in addition, work in non-aseptic conditions [115]. [91]Non-sterile fermentation can be a potential strategy to lower the costs associated with fermentation processes [116]. Singh et al. observed an increase of 10% in carotenoid production by *R. toruloides* when using 5% NaCl [108, 117]. Temperature and pH also influence carotenoid compound production. For instance, Da Silva et al. increased carotenoids production titers in *Rhodotorula mucilaginosa* by 3.3 times (from 0.34 g/L to 1.13 g/L) after reducing the cultivation temperature from 34 °C to 22 °C [104]. These authors also reported the benefit of lowering pH from 7.0 to 5.0 to favour carotenoid production.

The great potential of carotenoids in the biotechnology sector has stimulated developments in the industrialisation of cultures and genetic modification technologies. Park et al. reviewed different approaches to genetic engineering in *R. toruloides* in order to explore its full potential as a biotechnological platform for the bioproduction of industrially relevant compounds [118]. In this sense, the development of synthetic and system biology tools in this yeast species has increased our knowledge about its genomic organisation, metabolic pathways and their regulation, and the generation of essential genetic components (e.g., promoters, markers, terminators) required to advance genetic engineering approaches.

**Table 2:** Carotenoids produced by non-conventional oleaginous yeast from lignocellulosic biomass

Microorganism	Bio-product	Concentration/content	Raw material	Reference
*R. mucilaginosa*	Carotenoids	1.13 mg/L	Sisal bagasse hydrolysate (121ºC, 3 h Sulphuric acid solution (5.5%) S/L ratio 1:10	[104]
*R. pacifica INDKK*	β -carotene	210.4 mg/L	5% (v/v) molasses supplemented with enzymatically hydrolysis alkali-pretreated sugarcane bagasse hydrolysate (35% v/v).	[113]
*R. toruloides* ATCC 204091	β-Carotene	62 mg/L 57 mg/L	Waste ‘extract’ from fruit peels and inedible/discarded parts of vegetables as culture medium	[119]
*R. toruloides CBS 14*	Carotenoids	1.99 mg/100 dry cell weight	Wheat straw Acid-soaked (1% acetic acid) steam explosion 190 ºC/Enzymatic hydrolysis with cellulases	[80]
*R. toruloides CBS 14*	β-Carotene	1.48 mg/100 dry cell weight	Wheat straw hydrolysate (steam explosion 190ºC 10 min; 1% acetic acidic (soaked overnight)	[80]
*R. toruloides* DSM 4444	β-Carotene	16 mg/mL	*Camelina* meal as carbon source (SSF)	[120]
*R. toruloides NRRL Y-1091* *(evolved by adaptive laboratory evolution*	carotenoids	14.09 mg/g dry cell weight	Wheat straw, hydrothermal pretreatment (195º-45 min)	[91]
*R. toruloides NRRL Y 1091*	carotenoids	15 mg/L	Switchgrass (Deep eutectic solvent pretreatment	[111]
*R. toruloides RP15*	carotenoids	12.7 mg/L	Sugarcane bagasse (Diluted acid pretreatment)	[112]

Enzymes (e.g., lipases and endo-β-glucanases), bioethanol, and biosurfactants can also be co-produced during lipid accumulation as value-added compounds for increased process cost-effectiveness. Pi et al. engineered the oleaginous red yeast *R. glutinis* to simultaneously produce β-carotene (up to 27.13 mg/g) and cellulase enzymes without affecting the ability of the microorganism to accumulate lipids and to tolerate relatively high salt concentrations [121]. Cai et al. suggested an integrated biorefinery process to obtain microbial oil and bioethanol from corncob bagasse [122]. These authors used the liquid fraction obtained after dilute acid pretreatment (0.27wt % H_2_SO_4_, 120 ºC, 120 min, solid/liquid ratio 10%) for microbial lipid production, while the enzymatic hydrolysate resulting from the solid fraction was used for bioethanol production. This processing strategy resulted in 131.3 g of bioethanol/kg raw material and 11.5 g of microbial lipids/kg raw material using the yeasts *Saccharomyces cerevisiae* and *R. glutinis*, respectively. Brandenburg et al. studied the possibility of co-producing furfural, ethanol or lipids using wheat straw as the raw material [123]. The lignocellulosic biomass was pretreated by a patented thermochemical method. For ethanol production, they used the yeast *S. cerevisiae*; while for lipids, they used the oleaginous yeast *R. babjevae.* From 1 kg of wheat straw, 110 g of furfural, 111 g of ethanol or 33 g of lipids were obtained [124]. In addition, *R. babjevae* produced considerable amounts of heptadecenoic acid and α and ɣ-linolenic acid. Deeba et al. recently proposed a novel integrated biorefinery strategy to convert sugarcane industry waste into biodiesel, β-carotene, animal feed and xylooligosaccharides [113]. Xylooligosaccharides are obtained from the liquid fraction collected after alkali pretreatment and enzymatic treatment with xylanases, while lipids and β-carotene are obtained through fermentation with the red yeast *Rhodotorula pacifica* INDKK. Overall, this strategy resulted in 11.8 g/L of lipids, 210.4 mg/L of β-carotene, 7.1 g of animal feed, and 20.6 g/L of xylooligosaccharides.

Biosurfactants are also useful microbial-based compounds that yeast, fungi and bacteria can obtain. These compounds exhibit amphipathic chemical structures, mainly anionic or neutral, although cationic biosurfactants contain amine groups. The hydrophobic moiety is a long-chain fatty acid, and the hydrophilic moiety can be a carbohydrate, cyclic peptide, amino acid, phosphate, carboxylic acid, or alcohol [125]. They can be classified according to their chemical composition as glycolipids, lipopeptides or lipoproteins, polymers, fatty acids, neutral lipids and phospholipids. Biosurfactants have numerous advantages over synthetic surfactants, including higher biodegradability, low toxicity, thermal stability, resistance to extreme pH values, and ionic strength [125, 126]. Araújo et al. evaluated the production of biosurfactants by *R. mucilaginosa* LPP5 using an acid-catalysed hydrolysate from brewer’s spent grain as substrate [127]. The resulting biosurfactant was anionic in nature (most likely a glycolipid-type biosurfactant) and showed functional properties to form stable emulsions under different conditions such as temperature, pH, and salinity.

Implementing microbial lipids production from lignocellulosic materials and industrial wastes on a large scale also requires simplifying and integrating the different steps of the process. Simultaneous Saccharification and Fermentation (SSF) has been one of the most significant advances involving lignocellulosic ethanol production. However, configuring this process demands further development concerning microbial lipid production. Consolidated Bioprocessing (CBP) –which combines all the necessary stages (i.e., enzyme production, pretreatment, enzymatic hydrolysis, and sugar fermentation) to transform lignocellulose into lipids in a single reactor– is another promising integrated process. During CBP processes, a single microorganism or a mixture of microorganisms provides all the enzymatic activities needed to pretreat and hydrolyse a biomass and convert solubilised sugars into the product of interest [128]. Although CBP processes must be investigated thoroughly to optimise lipid production under this configuration, some examples can already be found in the literature. For instance, Intasit et al. proposed a CBP strategy for lipid production by co-cultivating the oleaginous fungus *Aspergillus tubingensis* TSIP 9 and different oleaginous yeasts (*Trichosporonoides spathulata* JU4-57, *Candida tropicalis* X37, *R. mucilaginosa* G43 and *Y. lipolytica* TISTR 5151) [129]. The highest lipid concentration and yield were obtained by combining *A. tubingensis* and *Y. lipolytica*, yielding 149.3 mg/g of palm empty fruit bunch.

Similarly, Doan et al. proposed the combination of the oleaginous fungus *Aspergillus orzyae* 32 and the oleaginous yeast *L. starkeyi* as a CBP system, reaching a lipid accumulation of 10.9 g/g of lime-pretreated rice straw (representing 8.5 g/100 g of raw rice straw) [130]. A single culture CBP strategy to produce microbial lipids from grasses has been investigated by Chuengcharoenphanich et al. using the cellulolytic oleaginous yeast *Cyberlindbera rhodanensis* CU_CV7 combined with an alkaline hydrogen peroxide pretreatment [131]. Using this strategy, the highest lipid titer and yield were 1.01 g/L and 50 mg/g, respectively, using Napier (Lampang ecotype) forage grass. Another interesting strategy combining SSF and CBP processes has been investigated by Zhao et al. These authors subjected a mixture of alkali-pretreated corn stover and cassava starch to fermentation with *L. starkeyi*, reporting a synergistic effect in lipid production using a lower enzyme dose, compared to using these substrates separately [132] .

## Future perspectives

**Fig. 2:**
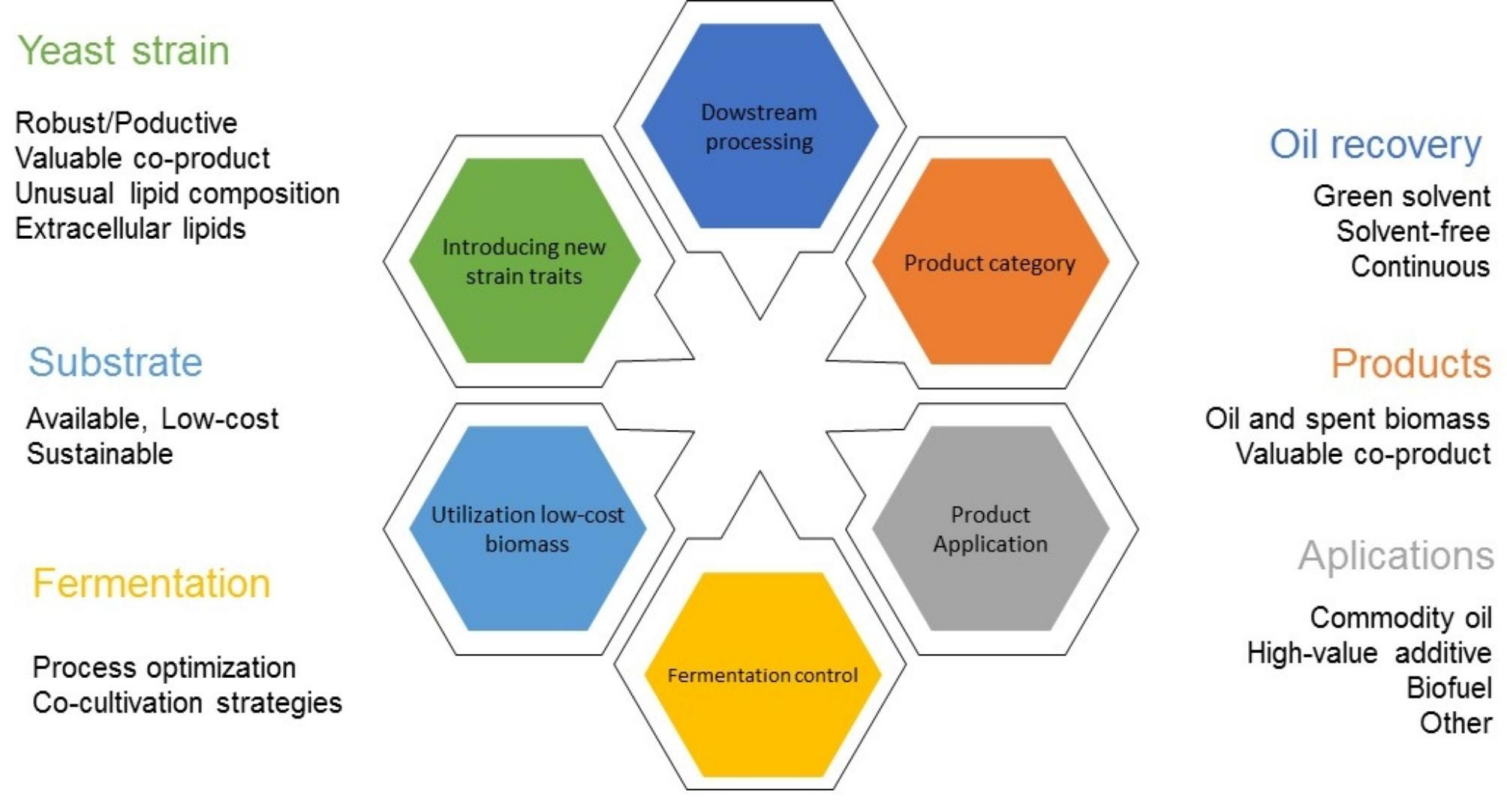
Important aspects requiring further investigation towards reaching cost-effective microbial oil production

In recent years, producing lipids from low-cost substrates such as lignocellulosic biomass and industrial wastes has been researched intensively, highlighting the importance of these conversion processes. Figure 2 identifies the main aspects of the lipid production processes and summarises the major challenges that must be investigated further. An essential aspect that requires further research before scaling up SCO production processes from lignocellulosic feedstocks and industrial wastes is the increase in lipid yield and volumetric productivity values. The presence of biomass-derived inhibitory compounds in the corresponding hydrolysates is currently an important limiting factor that needs more attention. Developing new strains capable of degrading and/or tolerating these inhibitors at higher concentrations might pave the way towards cost-effectiveness. In addition to evolutionary engineering strategies, targeted metabolic engineering using system and synthetic biology approaches will contribute to obtaining new microbial strains with increased robustness against inhibitory compounds and higher productivity values. In addition, these engineering techniques may also contribute to the design of novel metabolic pathways to obtain valuable co-products, an unusual lipid composition with industrial applications and/or easy lipid recovery by secretion of these compounds. Novel genome editing tools recently developed, such as the CRISPR/Cas9, system, are helping to achieve this goal by introducing genetic changes in microbial strains whose genomes were previously difficult to edit.

Process optimisation is also an important element to consider during microbial oil production. In this sense, it is important to improve further the cultivation strategy by selecting the best bioreactor type (e.g., stirred tank, airlift/bubble columns), working with high cell densities, finding the appropriate substrate feeding system during the accumulation phase (e.g., pulse-feeding, two-batch and continuous feeding strategy), and evaluating effective co-cultivation methods allow increased total lipid production and maximising co-product recovery. Optimisation of process parameters, including media composition, pH, temperature and aeration rate, is also crucial. The use of new technology using big data analytics and/or machine learning algorithms might contribute towards process optimisation. Finally, downstream processing also demands further research efforts in order to develop and implement novel methods for product recovery, including the use of solvent-free or green solvent methodologies to improve the sustainability of the overall process scheme. These downstream processing technologies must also be targeted at developing new processes allowing the continuous treatment of cell biomass and harvesting the product of interest.

## Conclusions

Using low-cost substrates becomes essential in making SCO competitive from an economic perspective. In addition, the co-production of value-added compounds with biotechnological potential within an integrated biorefinery concept will significantly contribute to cost-competitiveness during microbial lipid production from biomass residues. Research efforts have helped to develop different process strategies to increase SCO production using native oleaginous yeast strains. Still, implementing microbial lipids production from large amounts of lignocellulosic materials and industrial wastes requires simplifying and integrating the different process steps to deploy their full potential.

## Data Availability

Not applicable.
